# Pyruvic oxime dioxygenase from heterotrophic nitrifier *Alcaligenes faecalis* is a nonheme Fe^(II)^-dependent enzyme homologous to class II aldolase

**DOI:** 10.1038/srep39991

**Published:** 2017-01-06

**Authors:** Shuhei Tsujino, Chisato Uematsu, Hideo Dohra, Taketomo Fujiwara

**Affiliations:** 1 Department of Science, Graduate School of Integrated Science and Technology, Shizuoka University, Shizuoka 422-8529, Japan; 2 Department of Biological Sciences, Faculty of Science, Shizuoka University, Shizuoka 422-8529, Japan; 3 Instrumental Research Support Office, Research Institute of Green Science and Technology, Shizuoka University, Shizuoka 422-8529, Japan; 4 Department of Environment and Energy Systems, Graduate School of Science and Technology, Shizuoka University, Shizuoka 422-8529, Japan

## Abstract

Pyruvic oxime dioxygenase (POD), a key enzyme in heterotrophic nitrification, was purified from *Alcaligenes faecalis*, and the molecular and catalytic characteristics were reexamined. POD was purified as the homotetramer of the subunit whose molecular weight was 30,000. The deduced amino acid sequence of POD was homologous with a class II aldolase that has been regarded as the Zn^(II)^-dependent enzyme catalyzing aldol reactions. The recombinant protein showed weak POD activity, and was activated by reconstitution with Fe^(II)^. Affinity and catalytic constants were estimated at 470 μM and 4.69 sec^−1^, respectively. The POD was inactivated by EDTA to remove bound divalent metal cations. A reconstitution experiment demonstrated that Fe^(II)^, not Zn^(II)^, is essential for POD activity and that Mn^(II)^ could partially fulfill the function of Fe^(II)^. A mutant POD with replacement of His^183^, corresponding to one of three Zn^(II)^-binding ligands in the class II aldolase, by Asn was purified as a homotetrameric protein but showed no catalytic activities. Those results suggest that the POD is homologous to class II aldolase having non-heme Fe^(II)^ as a catalytic center instead of Zn^(II)^. A possible mechanism of the POD reaction is discussed on the basis of that of a known Fe^(II)^-dependent dioxygenase.

Nitrification is the microbial oxidation of ammonia to generate nitrate via nitrite, and is essential to the function of the nitrogen cycle in the environment. Several types of microbial ammonia oxidation processes with different biochemical mechanisms are known to occur: the first is an aerobic oxidation of ammonia by chemolithotrophic ammonia-oxidizing bacteria (AOB) and ammonia-oxidizing archaea (AOA), and the second is an anaerobic process by ANAMMOX bacteria. The biochemical mechanisms of ammonia oxidation in the microorganisms have already been elucidated, and their ecological characteristics are also under the active investigation^1^,^2^,^3^,^4^.

The third process of ammonia oxidation is driven by heterotrophic microorganisms; therefore, it has been designated heterotrophic nitrification. In early studies, it was already recognized that many kinds of heterotrophic soil microorganisms including bacteria and fungi possess the ability to oxidize ammonia to nitrite or nitrate^5^,^6^,^7^. The ammonia oxidizing activity in each cell of heterotrophic nitrifiers is lower than that of the autotrophic nitrifiers. However, probably due to the large biomass of heterotrophic microorganisms in the soil, it has been reported that the total rate of ammonia oxidation by heterotrophic nitrifiers might be comparable to that by autotrophic nitrifiers^8^. Heterotrophic nitrifiers are involved in the removal of nitrogen from farmland soils, and in addition have significance for application to wastewater treatment systems^9^,^10^. In spite of the apparent functional importance, exploring and understanding the biochemistry of the heterotrophic nitrification is still under way.

Aerobic ammonia oxidation by chemoautotrophic AOB is carried out through two sequential reactions involving ammonia monooxygenase (AMO) catalyzing the monooxygenase reaction of ammonia to form hydroxylamine (NH_2_OH), and hydroxylamine oxidoreductase (HAO) catalyzing the four-electron oxidation of NH_2_OH and generating nitrite^1^. AMO is an unstable membrane-bound protein, and HAO is a multiheme *c*, a high molecular weight enzyme^11^,^12^. It has been reported that the biochemical mechanism of ammonia oxidation in several heterotrophic nitrifying bacteria, *Arthrobacter globiformis and Paracoccus pantotropha*, is similar to the above-mentioned process of the AOBs. However, NH_2_OH oxidation is catalyzed by a nonheme Fe protein in both bacteria^13^,^14^.

Heterotrophic nitrification, a biochemical process different from the above-mentioned mechanism, has also been reported. In the mid-20^th^ century, the relevance of oxime and nitro compounds to nitrification in several heterotrophic microorganisms was reported^15^,^16^,^17^,^18^. *Alcaligenes faecalis* is a betaproteobacterium that has been commonly used for investigation of heterotrophic nitrification, and pyruvic oxime (2-(hydroxyimino)propanoic acid), and not its hydrolysis product, NH_2_OH, was found to be involved in the ammonia oxidation pathway of *A. faecalis*^6^. Yamanaka and his co-workers succeeded in purification of the enzyme that catalyzes oxygenation of pyruvic oxime to yield pyruvate and nitrite from the bacterium^19^,^20^. In the proposed mechanism of heterotrophic nitrification by *A. faecalis*, pyruvic oxime dioxygenase (POD) functions as the main character in combination with AMO as follows. In the first process, ammonia is oxygenized by AMO, then the resulting NH_2_OH thus generated is converted to pyruvic oxime by a non-enzymatic reaction with pyruvate^20^. Pyruvic oxime is degraded to pyruvate and nitrite during a dioxygenation reaction by POD^19^,^20^. POD was purified to an electrophoretically homogeneous state, and was demonstrated as a soluble, nonheme Fe-containing protein, although the DNA sequence of the enzyme was not determined^20^.

In this study, POD was purified from *A. faecalis* and the molecular and enzymatic properties were reexamined. The purified enzyme showed a homotetrameric configuration of the subunit whose molecular weight was 30,000. The DNA sequence of the gene encoding POD was determined. Surprisingly, the putative amino acid sequence of the enzyme was found to be homologous with that of class II aldolase, and three histidine residues for Zn^(II)^-binding were conserved. Reactivation of the recombinant protein by divalent metal cations demonstrated a requirement of Fe^(II)^ for the POD activity. A sequence alignment search and phylogenetic analysis suggested wide distribution of the putative POD genes among several microorganisms including proteobacteria, actinobacteria and eukaryotic fungi. A possible mechanism of the POD reaction is discussed on the basis of known Fe^(II)^-dependent dioxygenases.

## Results

### Purification of POD

*A. faecalis* was cultivated in a synthetic medium containing sodium pyruvate and ammonium as the only carbon and nitrogen sources, respectively. The highest POD activity was observed in the cells at the middle to late exponential growth phase; then, the activity decreased rapidly in the stationary phase. The nitrite concentration in the medium increased accompanying the growth of the cells, and finally reached 1.8 mM, which corresponded to 22% of the initial concentration of ammonium in the medium. *A. faecalis* cells were harvested in the exponential growth phase, then POD was purified from the cells according to the previous report with some modifications (Ono *et al*.^20^). The molecular weight of the purified POD was estimated as 30,000 by SDS-PAGE (data not shown).

### Identification of gene encoding POD

To determine the DNA sequence of the gene encoding POD, the *A. faecalis* genome was analyzed by next-generation sequencing. The draft genome of *A. faecalis* contained 27 contigs consisting of 4,042,912 bp with a G + C content of 56.66%. The draft genome sequence was annotated using Prokka version 1.11^21^ with an in-house bacterial database containing 5,399 bacterial genomes (Chromosome and Complete Genome only) and 6,649 plasmid sequences in the NCBI RefSeq database as of April 8, 2016^21^ and the annotations were manually curated. The annotated genome contained 3,719 protein-coding sequences and 53 tRNA genes. The genome also contained three rRNA operons, consistent with the number of rRNA operon of the *A. faecalis* strain ZD02 complete genome (accession no. NZ_CP013119), judging from the three-fold coverage of the contig (accession no. BDHG01000020) containing 5S, 16S, and 23S rRNA genes. The draft genome sequence of *A. faecalis* NBRC13111 has been deposited at DDBJ/EMBL/GenBank under the accession no. BDHG00000000 including sequences of 27 contigs (BDHG01000001-BDHG01000027).

**Figure 1:**
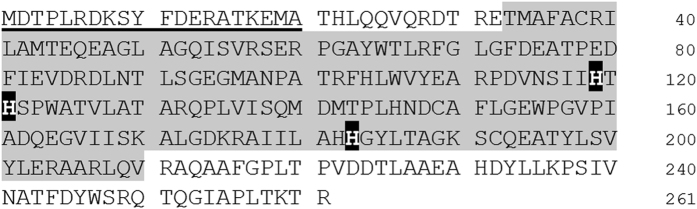
Putative amino acid sequence of *A. faecalis* POD. The gene encoding POD (GAU72725) was identified from the draft sequence of *A. faecalis* genomic DNA. The N-terminal amino acid sequence of the purified POD is underlined. The shaded part of the sequence was annotated as Class II aldolase and adducing N-terminal domain (pfam00596). Putative residues for metal-binding (His119, His121, His183) are emphasized by white characters.

The N-terminal sequence of the purified POD was determined as MDTPLEDKSYFDEXATXEMA (X could not be identified due to weakness of the signal). A sequence homology search against the draft genome data of *A. faecalis* identified a gene encoding a protein 261 amino acids long of which the 1^st^–20^th^ sequence was identical to the N-terminal sequence of the purified POD (Fig. 1). The gene product was expected to be a soluble and cytoplasmically localized protein whose molecular weight and isoelectric point were estimated as 29,106 and 5.04, respectively. An inert region (33^rd^–210^th^) of the putative amino acid sequence was annotated as a class II aldolase and adducin N-terminal domain (pfam00596). Generally, class II aldolase possesses Zn^(II)^, which is coordinated by three histidine residues as the reaction center. Corresponding histidines, His119, His121, and His183, were also conserved in the gene product (Fig. 1).

### Enzymatic characterization of recombinant POD

**Figure 2:**
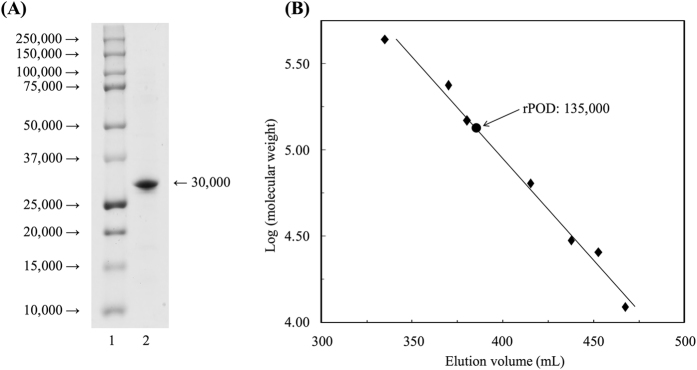
SDS-PAGE and gel filtration analyses of recombinant POD in purified state. **(A)** A purified sample treated with 2% SDS plus 2% β-mercaptoethanol and boiling (lane 2) and loaded onto 10% polyacrylamide gel. Lane 1 represents the standard proteins whose molecular weights were indicated on the left side of the gel. **(B)** The molecular weight of rPOD in the solution was determined by Sephacryl S-200 gel filtration using standard proteins as mentioned in Methods.

The recombinant POD (rPOD) was overexpressed in *Escherichia coli* and purified to electrophoretically to a homogeneous state as shown in Fig. 2. The purified preparation showed POD activity. The molecular weight of the rPOD was estimated to 135,000 by gel filtration, indicating a homotetrameric configuration in the solution (Fig. 2).

As indicated in Table 1, the specific activity of the purified rPOD was measured as 0.087 μmol/min/mg protein, and this value was much lower than that of the purified preparation (1.24 μmol/min/mg protein) reported by Ono *et al*.^20^. The reason for the low catalytic activity might be that the POD is unstable: the activity of rPOD decreased gradually during purification and storage even at 4 °C, similar to the enzyme prepared from the *A. faecalis* cells. The Fe content in the purified rPOD was also very low (0.03 Fe atoms/enzyme) compared with the value (2.2 atoms/enzyme) that had been reported previously^20^. On the other hand, purified rPOD was activated by supplementation of Fe^(II)^. The specific activity of rPOD, which had been incubated with 100 μM FeSO_4_, was determined as 3.02 μmol/min/mg protein (Table 1). Similar activity was observed in the rPOD that had been incubated with 10 μM FeSO_4_ (data not shown). After the reactivation of rPOD and successive dialyzation to remove unbound Fe^(II)^, the Fe content of the preparation was determined to be 0.54 ± 0.01 atoms/subunit (Table 1). The remaining activity of the preparation was determined to be 1.52 ± 0.24 μmol/min/mg protein, which was about 50% of that of the activated rPOD. These results suggested the stoichiometric composition of the holo-state POD as one Fe^(II)^ bound in one subunit molecule, but Fe^(II)^ might be released easily from the POD.

Enzymatic activity of the rPOD was completely lost by treating it with 1 mM EDTA. However, the activity of the EDTA-treated rPOD was recovered to the same level as that of the reactivated enzyme mentioned above by supplementation of 10–100 μM FeSO_4_ into the assay solution (Supplementary Fig. S1). Steady state kinetic analysis of the POD reaction was performed in the assay solution supplemented by 100 μM FeSO_4_. The affinity constant to pyruvic oxime (K_m_) and apparent catalytic constant (*k*_cat_) of the Fe^(II)^-reconstituted rPOD were estimated to be 470 μM and 4.69 sec^−1^ (per subunit molecule), respectively, according to Hanes-Woolf plotting. The POD activity was maximal at pH 8.0. POD activity was inhibited by cyanide ion; the half maximal inhibitory concentration (IC_50_) for cyanide was about 400 μM (Supplementary Fig. S2).

**Figure 3:**
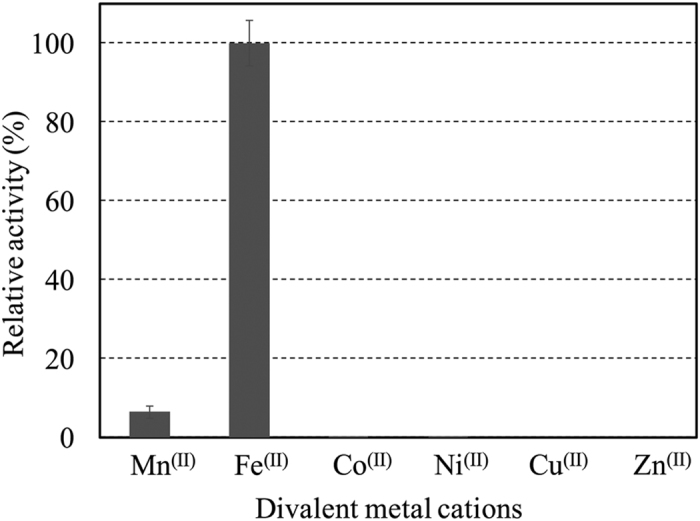
Reconstitution of rPOD by divalent metal cations. The rPOD, which was inactivated by EDTA-treatment and subsequent dialyzation to remove divalent metal cations including Fe^(II)^, was used for the reactivation experiment described in Methods. POD activity was measured in the assay solution containing 100 μM each of MnCl_2_, FeSO_4_, CoCl_2,_ NiCl_2_, CuCl_2_, or ZnSO_4_. No activities were detected before addition of metal cations (data not shown). Experiments were performed independently three times. Error bars represent S.E.

A reconstitution experiment with divalent metal species demonstrated that replacement of Fe^(II)^ by Mn^(II)^ was possible, while the Mn^(II)^-dependent POD activity was only 6.5% of that of the enzyme reconstituted by Fe^(II)^, as shown in Fig. 3. No POD activity was observed when the inactivated rPOD was reconstituted with Co^(II)^, Ni^(II)^, Cu^(II)^, or Zn^(II)^.

Site-directed mutagenesis on the *A. faecalis* POD was done to replace one of the three histidine residues that corresponded to Zn^(II)^-binding ligands in the class II aldolase. The mutant POD, in which the His183 was replaced to Asn, was purified to homogeneity and was confirmed to have a homotetrameric configuration (data not shown). The mutant enzyme did not show any POD activities even in the assay condition of supplementation with Fe^(II)^.

### Sequence alignment of POD with other class II aldolase

**Figure 4:**
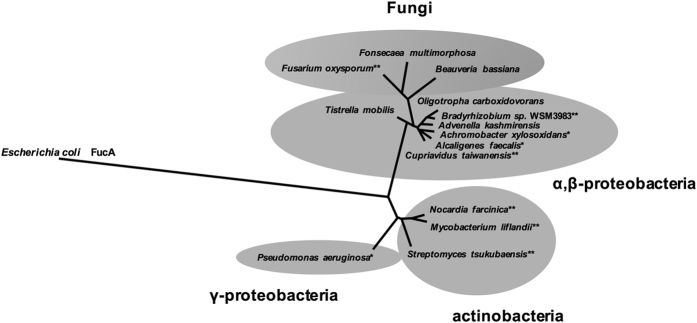
Phylogenetic relationship of *A. faecalis* POD with other putative PODs and *E. coli* FucA. Unrooted neighbor-joining tree drawn by the Phylogeny program was revealed using the amino acid sequences of putative PODs from *Achromobacter xylosoxidans* NBRC15126 (AX27061_2290, 84.7%), *Advenella kashmirensis* (TKWG_21375, 84.7%), *Cupriavidus taiwanensis* (WP_018007454.1, 84.3%), *Bradyrhizobium sp.* WSM3983 (WP_027535094.1, 79.7%), *Oligotropha carboxidovorans* OM4 (OCA4_c31890, 79.0%), *Tistrella mobilis* (TMO_b0354, 79.0%), *Beauveria bassiana* ARSEF2860 (XP_008599769.1, 62.4%), *Fusarium oxysporum* Fo5176 (EGU87391.1, 61.8%), *Fonsecaea multimorphosa* CBS102226 (KIY01105.1, 60.3%), *Streptomyces tsukubaensis* (WP_006345117.1, 49.4%), *Mycobacterium liflandii* (MULP_00998, 49.2%), *Nocardia farcinica* (CRY74867.1, 48.5%), and *P. aeruginosa* PAO1 (PA0224, 46.9%), and the sequence of *E. coli* FucA (AAA23823.1, 20.7%). Accession numbers and identities of amino acid sequence of *A. faecalis* POD are shown in parentheses. The species marked by single asterisks (*) have been reported to have heterotrophic nitrifying ability. Heterotrophic nitrifying ability has not been found in the microbes marked by double asterisks (**), but the different species involved in the same genera have been known as the heterotrophic nitrifiers.

The genes encoding the protein homologous to *A. faecalis* POD with 50–90% identities were found in several microorganisms included in phylum proteobacteria, phylum actinobacteria, and eukaryotic phylum ascomycota, which have been reported to have heterotrophic nitrifying abilities (Fig. 4). Molecular weights of the POD homologues were within a range around 30,000, and three histidine residues that are characteristic of the class II aldolase for Zn^(II)^-binding were also conserved. A sequence alignment search indicated that L-fuculose-phosphate aldolase (FucA), one of the members of class II aldolase whose physiological function is already known, showed the highest sequence similarity to POD; however, the amino acid sequence identity of *A. faecalis* POD with *E. coli* FucA was only 20.7%. Except for three histidines for Zn^(II)^-binding, none of the amino acid residues that are significant for recognition of the substrate (Asn29, Thr43, Gly44, Ser71, Ser72) and formation of the active center (Glu73, Tyr113, Gly132) in the *E. coli* FucA were conserved in *A. faecalis* POD^22^. Bacterial fructose 1,6-bisphosphatase (FBP) is also a class II aldolase, but the sequence similarity to POD was even lower. The rPOD revealed no FBP activity when the protein was reconstituted by Zn^(II)^ or other divalent metal cations.

## Discussion

In this study, the molecular mechanism of heterotrophic nitrification in *A. faecalis* was reexamined. POD was purified, and the molecular and catalytic properties reported previously were confirmed. Ono *et al*.^20^ have suggested that POD is a homotrimer of the subunit molecule whose molecular weight is about 40,000; however, a homotetrameric configuration of POD consisting of the subunit whose molecular weight was 30,000 was ascertained after our reconsideration.

In this study, the gene encoding POD was identified in the *A. faecalis* genome DNA, and its putative amino acid sequence was determined. Interestingly, POD was found to be homologous to class II aldolase (Fig. 1). Class II aldolase is the enzyme catalyzing cleavage of carbon-carbon bonding or the reverse by the aldol reaction using Zn^(II)^ as the active center. In contrast, as previously pointed by Ono *et al*.^20^, nonheme Fe was found to be contained in the purified POD. The reactivation and reconstitution experiments shown in Fig. 3 clearly demonstrated that the POD required Fe^(II)^, not Zn^(II)^, for the catalytic activity.

Although the catalytic activity of POD readily decreased with the removal of Fe^(II)^, the stoichiometric composition with one Fe^(II)^ in a subunit molecule was expected in the holo-state POD, as shown in Table 1. The Fe^(II)^-binding manner in the POD is not understood; however, it is probable that Fe^(II)^ is coordinated by the N atoms of three histidines corresponding to a conserved Zn^(II)^-binding site of the class II aldolase. The expectation seems to be consistent with the result that the rPOD with His183 → Asn mutation showed no POD activity.

**Figure 5:**
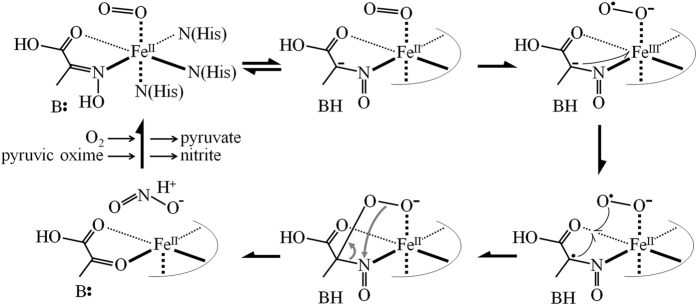
Possible reaction mechanism of POD. See the text for further details.

The molecular process of the POD reaction was not identified at this time, but mechanistic insight can be obtained from that of a known Fe^(II)^-dependent dioxygenase. Catechol 2,3-dioxygenase (EC1.13.11.2), catalyzing an extradiol-type ring cleavage of catechol, is an Fe^(II)^-dependent dioxygenase whose structure and reaction mechanism are well understood^23^,^24^. On the basis of the putative reaction mechanism of catechol 2,3-dioxygenase, an enzyme-substrate complex of the POD’s active center was postulated as an Fe^(II)^ octahedrally coordinated by three N atoms of corresponding histidines, a carboxylic O atom, and an oxime N atom of pyruvic oxime, and dioxygen molecule, as shown in Fig. 5. In the next step, deprotonation of pyruvic oxime by a base should occur to form a negative charge on the β-carbon (Cβ). A one electron reduction of dioxygen and formation of superoxo-Fe^(III)^ is also essential for the dioxygenase reaction. Fe^(III)^ is re-reduced to Fe^(II)^ by the equivalent electrons supplied from negatively charged Cβ. Finally, a radical-radical coupling between the superoxide and Cβ of pyruvic oxime for C-O bond formation and subsequent isomerization leads to generation of pyruvate and nitrite (Fig. 5). The Mn^(II)^-dependent reactivation observed in POD has also been reported in homoprotocatechuate 2,3-dioxygenase, a homologous enzyme to catechol 2,3-dioxygenase, suggesting a similar mechanistic behavior between the enzymes^25^.

As shown in Fig. 4, a sequence alignment search demonstrated that the gene encoding a putative POD was identified in the several proteobacteria that have been reported to be capable of heterotrophic nitrification^6^,^17^,^26^,^27^,^28^. The genes were also found among actinobacteria and an eukaryotic microorganism, ascomycota, in which the heterotrophic nitrifying ability has been previously indicated^5^,^7^. The sequences of POD homologous protein from ascomycota showed similarity to those of alfa and betaproteobacteria, whereas POD from actinobacteria showed a close phylogenetic relationship with POD from *Pseudomonas aeruginosa*, which belongs to the phylum gammaproteobacteria. It is notable that generation of nitrite from acetoaldoxime, not from pyruvic oxime, was reported in *P. aeruginosa*, suggesting a diversity of substrate specificity among the PODs^17^. To confirm the function of these homologous proteins, enzymatic analysis of the recombinant proteins is now in progress.

In this study, we demonstrated that POD from heterotrophic nitrifying bacterium *A. faecalis* was an Fe^(II)^-dependent enzyme whose sequence is homologous to that of class II aldolase. Class II aldolase was originally known to be a Zn^(II)^-dependent enzyme catalyzing the aldol reaction in the glycolysis or gluconeogenesis pathways of the microorganisms. The present finding is suggestive in terms of the molecular evolution of the metal enzyme because it raises the possibility that a protein homologous to class II aldolase was converted to dioxygenase by replacement of the Zn^(II)^ center with Fe^(II)^. A gene encoding the class II aldolase whose enzymatic activity has not been understood is often found in several microbial genomes. These class II aldolase-like proteins might function as an oxygenase or other enzyme rather than an aldolase in microbial metabolic processes. In addition, this finding is expected to be applicable to ecological investigation of the microbial nitrogen cycle. Quantitative analysis of the ecological distribution and population of at least a subset of the heterotrophic nitrifying microbes will become possible by direct detection of POD genes from environment samples.

## Methods

### Cultivation of A. faecalis

The medium for cultivation of *Alcaligenes faecalis* NBRC13111^T^ contained 10.8 g/L K_2_HPO_4_, 0.53 g/L KH_2_PO_4_, 0.428 g/L NH_4_Cl, 0.20 g/L MgSO_4_·7H_2_0, 38.9 mg/L CaCl_2_, 24.2 mg/L Na_2_MoO_4_·2H_2_O, 5.6 mg/L FeSO_4_·7H_2_O, 0.99 mg/L MnCl_2_·4H_2_O and 1.1 g/L sodium pyruvate, and was adjusted to pH 8.0 before autoclaving. After inoculation of the preculture (100 mL) into 4 L of the medium, *A. faecalis* was cultivated in the medium using a fermenter (model MD-300, Marubishi Co., Ltd., Tokyo, Japan) at 25 °C with vigorous aeration. When the optical density of the medium at 600 nm (OD_600_) reached 0.6, the microbial cells were collected by centrifugation, and used for purification of POD.

### Purification of POD

Purification of the POD was carried out according to Ono *et al*.^20^ with some modifications, as mentioned in the Supplementary Materials. POD activity was determined by measuring the rate of nitrite production in the assay solution containing 10 mM Tris-HCl buffer (pH 8.0), 1 mM sodium ascorbate, and 1 mM pyruvic oxime. The reaction was started by mixing 5 μL samples with 2 mL of the assay solution. The solution was incubated at 37 °C for appropriate periods, then the nitrite concentration in the assay solution was determined spectrophotometrically by a diazo-coupling method^29^.

### Identification of gene encoding POD

N-terminal amino acid sequences of the purified POD were determined as follows: the purified preparation was subjected to SDS-PAGE^30^, then the protein band with an estimated molecular weight of 30,000 in the gel was transferred to a polyvinylidene difluoride (PVDF) membrane (Millipore, Bedford, MA, USA) by a semi-dry electroblotting device (ATTO Co., Tokyo, Japan). The bands on the PVDF membrane were subjected to a PPSQ-21 protein sequencer (Shimadzu Co., Kyoto, Japan) to determine the N-terminal sequence. The gene encoding the protein whose N-terminal sequence was identical to that of the purified POD by a sequence alignment search on the draft data of the *A. faecalis* genome. Determination of the draft genome sequence is described in the Supplementary Materials.

### Construction of POD expression vector

According to the DNA sequence of the putative POD gene, oligonucleotide primers for PCR amplification of the gene encoding *A. faecalis* POD, AfpodF (5′-CCA TAT GGA TAC CCC ACT CAG AGA-3′, artificial *Nde*I recognition site underlined) and AfpodR (5′-GCT CGA GTC AGC GAG TTT TAG TTA AGG GCG-3′, artificial *Xho*I site underlined), were designed. Standard protocols used for DNA handling in *E. coli* followed Sambrook and Russell^31^. Amplification was carried out using KOD-plus DNA polymerase (Toyobo, Osaka, Japan) and *A. faecalis* genomic DNA as a template. The 783 bp PCR product obtained was cloned into a pCR-blunt TOPO II vector (Invitrogen, Carlsbad, CA, USA), yielding pCRAfPOD. After confirmation of the nucleotide sequence, the insert of pCRAfPOD was digested with both *Nde*I and *Xho*I, and then cloned into the same restriction site of a pET21a^+^ expression vector (Novagen, Darmstadt, Germany), yielding the expression plasmid pAfPOD. The pAfPOD plasmid was introduced into *E. coli* BL21(DE3)-CodonPlus (Agilent Technologies, Santa Clara, CA. USA), generating strain AfP01 for overexpression of recombinant POD (rPOD).

### Purification of recombinant POD

Strain AfP01 was cultivated aerobically in the 2×YT medium (1 L) which was supplemented with 50 µg/mL ampicillin at 37 °C with reciprocal shaking at 150 rpm. In a mid-exponential growth stage (OD_600_ = 0.6–0.8), 0.1 M stock solution of isopropyl β-D-1-thiogalactopyranoside (IPTG) was added to the medium to reach 0.3 mM for induction of rPOD. After incubation at 20 °C with shaking at 150 rpm for 3 h, the cells were collected by centrifugation. Cultivated AfP01 cells were suspended in 40 mL of 20 mM Tris-HCl (pH 8.0) containing and 10 μM phenylmethylsulfonyl fluoride (PMSF) (buffer A). The suspension was sonicated using a VP-30S supersonic oscillator (Taitec Co., Ltd, Saitama, Japan) for 30 × 10 sec at full power to disrupt cells. After removing unbroken cells by centrifugation at 12,000 × *g* for 10 min, the supernatant obtained was centrifuged at 140,000 × *g* for 65 min using an Optima L-90K ultracentrifuge (Beckman Coulter, Inc., Brea, CA, USA). The soluble fraction thus obtained was applied to an anion-exchange chromatography column (2 × 12 cm) of DEAE-Toyopearl 650 M gel (Tosoh, Tokyo, Japan) that had been equilibrated with buffer A. The recombinant protein adsorbed on the column was eluted by a linear gradient generated from 100 mL each of buffer A and buffer A containing 0.4 M NaCl. The fractions that showed POD activity were collected, then concentrated by 30–50% saturated (NH_4_)_2_SO_4_ fractionation. The precipitant obtained was suspended in 1 mL of buffer A containing 0.25 M NaCl, then applied to a column (3 × 110 cm) of Sephacryl S-200 (GE Healthcare, Little Chalfont, UK) that had been equilibrated with the same buffer. The fractions showing POD activity were collected, then concentrated again by (NH_4_)_2_SO_4_ fractionation as above. The precipitant was suspended in a minimal volume of 20 mM Tris-HCl buffer (pH 8.0) and further concentrated centrifugally using an Amicon Ultra Centrifugal Filter Unit 50k (Millipore), and used as the purified sample for experiments.

### Molecular and enzymatic analysis

The molecular weight of rPOD was determined by Sephacryl S-200 gel filtration using ferritin (molecular weight: 440,000), bovine liver catalase (240,000), alcohol dehydrogenase (150,000), hemoglobin (64,500), carbonic anhydrase (30,000), α-chymotrypinogen (25,700), and horse mitochondrial cytochrome *c* (12,500) as standard proteins.

Activation of the purified rPOD was carried out by incubation in the 20 mM Tris-HCl buffer (pH 8.0) containing 1 mM sodium ascorbate and 100 μM FeSO_4_ for 1 h at 4 °C. The resulting solution (about 1 mL) was dialyzed two times against 1 L of 20 mM Tris-HCl buffer (pH 8.0) for 1 h at 4 °C to remove unbound Fe^(II)^. Apparent POD activities of the purified, activated, and dialyzed preparations thus obtained were measured in the assay solution described above. Fe concentrations of the three samples were also determined by using a Nitroso-PSAP assay kit (Metallogenics, Chiba, Japan).

The apparent rate constant (*k*_cat_) and affinity constant (K_m_ for pyruvic oxime) were determined according to Hanes-Woolf plots in the assay solution containing 100 μM FeSO_4_. The effect of pH on the POD activity was analyzed by measuring the activity of the purified preparation in the assay solution containing 100 μM FeSO_4_, of which the pH was adjusted by a wide range buffer (pH 6.5–9.5, containing 5 mM each of Tris-base, MOPS, and glycine) instead of Tris-HCl buffer. The IC_50_ of cyanide was determined in the assay solution supplemented with potassium cyanide.

The rPOD was inactivated by dialysis against 20 mM Tris-HCl buffer (pH 8.0) containing 1 mM disodium ethylenediamine tetraacetate (Na_2_-EDTA) for 1 h to remove divalent metal cations bound to the enzyme molecule. The resulting solution (about 1 mL) was dialyzed two times against 1 L of 20 mM Tris-HCl buffer (pH 8.0) for 1 h at 4 °C to remove EDTA.

Reconstitution of inactivated rPOD with Fe^(II)^ and other divalent metal cations was evaluated by measuring the apparent POD activities in the assay solution supplemented by 100 μM FeSO_4_. Reconstitution experiments were also carried out using other divalent metal cations: the POD activity was determined in the assay solution containing 100 μM MnCl_2_, CoCl_2,_ NiCl_2_, CuCl_2_, or ZnSO_4_ instead of FeSO_4_.

### Point mutation

Site-directed mutagenesis of the recombinant POD was carried out by technical application of PCR. One set of oligonucleotide primers, AfPODH183Nf (5′-GCG CCA TTA TTC TGG CCC ACA ATG GTT ATC TGA CCG CAG GCA A-3′, position of mutation is underlined) and AfPODH183Nr (5′-TTG CCT GCG GTC AGA TAA CCA TTG TGG GCC AGA ATA ATG GCG C-3′, position of mutation is underlined), in which the corresponding His183 (CAC) was replaced with Asn (AAT), were amplified by using the pAfPOD plasmid as a template. After treatment with restriction enzyme *Dpn*I to decompose the template DNA, the PCR product was introduced into the *E. coli* JM109 cells. The PCR product was cyclized by homologous recombination between the 5′ and 3′ regions in the host cells. The resulting plasmid, pAfPODH183N, for over-expression of the mutant POD was introduced into *E. coli* BL21(DE3)-CodonPlus, generating strain AfP-2.

### Other experiments

Pyruvic oxime was synthesized according to Quastel *et al*.^16^. The protein concentration was measured using a BCA protein assay kit (Pierce, Rockford, IL, USA) with bovine serum albumin as the standard. Spectroscopic analysis in the visible region was carried out in a 1 cm light-path cuvette using an UV-2600 spectrophotometer (Shimadzu). Homology search and phylogenetic analysis were performed using Blast (http://blast.genome.jp/) and Phylogeny (http://www.phylogeny.fr/), respectively. Fructose 1,6-bisphosphatase (FBP) activity of the rPOD that had been reconstituted with divalent metal cations was determined in the assay solution containing 3 μM MnCl_2_, FeSO_4_, CoCl_2,_ NiCl_2_, CuCl_2_, or ZnSO_4_, according to the previous method^32^. All chemicals used in the experiments were of the highest grade commercially available.

## Additional Information

**How to cite this article**: Tsujino, S. *et al*. Pyruvic oxime dioxygenase from heterotrophic nitrifier *Alcaligenes faecalis* is a nonheme Fe^(II)^-dependent enzyme homologous to class II aldolase. *Sci. Rep.* **7**, 39991; doi: 10.1038/srep39991 (2017).

**Publisher's note:** Springer Nature remains neutral with regard to jurisdictional claims in published maps and institutional affiliations.

## Supplementary Material

Supplementary Materials

## Figures and Tables

**Table 1:** Specific activity and Fe content of the recombinant POD.

	Specific activity (μmol/min/mg protein)	Ratio of Fe atoms by subunit (mol/mol)
Purified	0.087	0.03
Reactivated	3.02	1.1 (estimated)
Dialyzed	1.52 ± 0.24	0.54 ± 0.01

Specific activity and Fe content of the rPOD in the purified, reactivated, and dialyzed states were determined. Reactivation of the purified rPOD was carried out by adding 100 μM FeSO_4_ to the sample as described in Materials and Methods. After dialyzation of the reactivated preparation to remove unbound Fe^(II)^, Fe concentrations of rPOD was determined by three individual measurements. The molecular weight value of the subunit of 30,000 was used to estimate the ratio of Fe atoms by subunit (mol/mol). The result is indicated with mean ± standard error (S.E.).
