# Supplementary material for: Pyruvic oxime dioxygenase from heterotrophic nitrifier Alcaligenes faecalis is a nonheme Fe(II)-dependent enzyme homologous to class II aldolase
Source: Sci Rep. 2017 Jan 6;7:39991. doi: 10.1038/srep39991 (PMC5216522; doi:10.1038/srep39991)
Supplement: Supplementary Materials [file srep39991-s1.pdf]

Supplementary materials (SREP-16-34780A)

Pyruvic oxime dioxygenase from heterotrophic nitrifier *Alcaligenes faecalis* is a nonheme $Fe^{(II)}$-dependent enzyme homologous to class II aldolase

Shuhei Tsujino[1], Chisato Uematsu[2], Hideo Dohra[3], and Taketomo Fujiwara[4*]

[1]Department of Science, Graduate School of Integrated Science and Technology,
[2]Department of Biological Sciences, Faculty of Science,
[3]Instrumental Research Support Office, Research Institute of Green Science and Technology,
[4]Department of Environment and Energy Systems, Graduate School of Science and Technology,
Shizuoka University, Shizuoka 422-8529, JAPAN

SUPPLEMENTARY MATERIALS

Methods

Purification of POD

Cultivated *A. faecalis* cells were disrupted two times using an Emulsifies C5 high pressure homogenizer (Avestin Inc., Ottawa, ON, Canada) at 12,000 psi. The resulting lysate was centrifuged at 12,000×g for 10 min to remove unbroken cells and debris. The supernatant that showed POD activity was mixed with fine $(NH_4)_2SO_4$ crystals to 30% saturation. After gentle stirring on ice for 30 min, the solution was centrifuged at 12,000×g for 10 min to remove precipitate. The supernatant was further mixed with $(NH_4)_2SO_4$ to 70% saturation, then was centrifuged again under the same condition. The precipitant obtained was suspended in 50 mL of 10 mM HEPES buffer (pH 7.0) (buffer B). After dialysis against buffer B, the sample was applied to an anion-exchange chromatography column (2×10 cm) of DEAE-Toyopearl 650M gel (Tosoh) that had been equilibrated with buffer B. The proteins adsorbed on the column were eluted by a linear gradient generated from 100 mL each of the buffer B and the buffer B containing 0.3 M NaCl. The fractions that showed POD activity were collected, and precipitated by 30%-60% saturated $(NH_4)_2SO_4$ fractionation. The pelleted material obtained was suspended in a minimal volume of 10 mM Tris-HCl buffer (pH 8.0) (buffer C), then applied to a column (2×15 cm) of Sepharose CL-4B (GE Healthcare) that had been equilibrated with the buffer C containing 60% saturated $(NH_4)_2SO_4$. The proteins adsorbed on the column were eluted with a linear gradient generated from 200 mL each of the buffer C containing 60% saturated $(NH_4)_2SO_4$ and the buffer C. The POD typically eluted in the fraction containing about 30% saturated $(NH_4)_2SO_4$. The fractions showing enzymatic activity were corrected, dialyzed against the buffer C, concentrated using concentrated using a Microcon YM-30 (Millipore), and used for the experiments.

Determination of draft genome sequence of **A. faecalis**

The genomic DNA of *A. faecalis* was isolated using a DNeasy blood and tissue kit (Qiagen, Hilden, Germany) and fragmented using the Covaris Acoustic Solubilizer. A paired-end library (2 × 301 bp) constructed

by the TruSeq DNA PCR-Free Library Preparation Kit (Illumina) was sequenced using the Illumina MiSeq platform at the Instrumental Research Support Office, Research Institute of Green Science and Technology, Shizuoka University. The raw read sequences were cleaned up using Trimmomatic[1] by trimming adapter sequences and low-quality ends (quality score, <15) and khmer[2] by filtering the reads with a low k-mer coverage (<5) to remove sequencing errors and contamination sequences. The resultant 1,251,216 high-quality reads totaling 327.16 Mb, which corresponds to an approximately 81-fold coverage of the genome, were assembled using SPAdes version 3.8.0[3] with k-mer sizes of 21, 33, 55, 77, 99, and 127 bp with options (--careful, --only-assembler, and --cov-cutoff auto), and contigs less than 200-bp were eliminated. The sequence data have been archived at DDBJ Sequence Read Archive (DRA) under the accession no. DRA005070.

1. Bolger, A. M., Lohse, M. & Usadel, B. Trimmomatic: a flexible trimmer for Illumina sequence data. *Bioinformatics* **30**, 2114-2120 (2014).

2. Crusoe, M. R., *et al*. The khmer software package: enabling efficient nucleotide sequence analysis. F1000Res **4**, 900 (2015) 10.12688/f1000research.6924.1.

3. Bankevich, A., *et al*. SPAdes: a new genome assembly algorithm and its applications to single-cell sequencing. *J. Comput. Biol.* **19**, 455-477 (2012) 10.1089/cmb.2012.0021

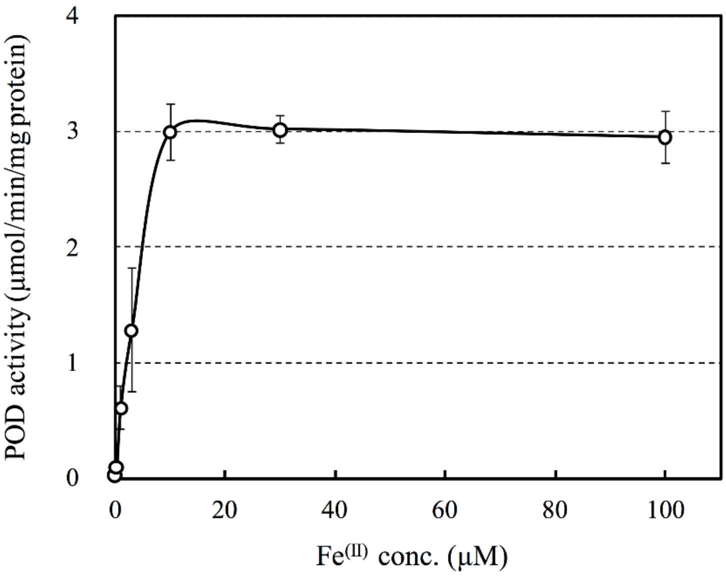

Supplementary Figure S1. $Fe^{(II)}$-dependent activation of rPOD. The rPOD inactivated by EDTA-treatment was reactivated as described in Materials and Methods. POD activity was measured in the assay solution containing several concentrations of $FeSO_4$. Experiments were performed independently for three times. Error bars represent S.E.

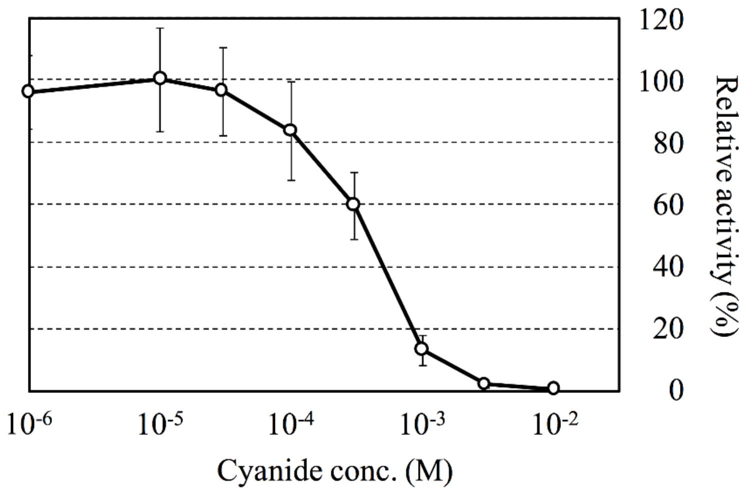

Supplementary Figure S2. Inhibition of POD activity by cyanide. Inhibitory effect of cyanide on the activity of the rPOD which was reconstituted by $Fe^{(II)}$ was measured as mentioned in Materials and Methods. Experiments were performed independently for three times. Error bars represent S.E.
